# Impact of Situational Variables on Goal-Scoring Offensive Sequences in the 2022 FIFA World Cup

**DOI:** 10.5114/jhk/202563

**Published:** 2025-09-23

**Authors:** Carlos Humberto Almeida, Paulo Paixão, António Jorge, Pedro Vargas, Ricardo Gonçalves, Rui Batalau

**Affiliations:** 1 Research Group in Soccer and Futsal (GIFut), Department of Psychology and Physical Education, Manuel Teixeira Gomes Higher Education Institute (ISMAT), Portimão, Portugal.; 2 Research Center in Sport, Physical Education, Exercise and Health (CIDEFES), Lusófona University (UL), Lisbon, Portugal.; 3 Department of Arts, Humanities and Sport, School of Education, Polytechnic Institute of Beja, Beja, Portugal.; 4 SPRINT: Sport, Physical activity and health Research & INnovation cenTer, Polytechnic Institute of Beja, Beja, Portugal.; 5 Jean Piaget Algarve School of Health, Piaget Institute, Silves, Portugal.

**Keywords:** soccer, notational analysis, match context, team performance, technical component

## Abstract

Research in match performance analysis has progressed markedly, yet detailed studies on technical-tactical indicators, notably those addressing multiple situational variables, remain sparse. To narrow this literature gap, this study aimed to examine the impact of four situational variables (competition stage, match period, match status, and team quality) on key performance indicators (KPIs) in goal-scoring sequences during the 2022 FIFA World Cup. All 168 goal-scoring sequences from regular time were evaluated post-event using the Offensive Sequences Characterisation System, which included simple and composite indicators. Another three categorical variables (ball recovery type, ball recovery location, and team possession type) were also coded. To evaluate the effects of situational variables, Mann-Whitney U and Kruskal-Wallis tests were applied to KPIs, whilst Chi-square and Multinomial Logistic Regression were conducted for categorical variables. The analysis revealed that while competition stage, match period, and match status did not significantly affect KPIs related to build-up and progression, they noticeably influenced defensive-to-offensive transitions, particularly during mid-game (31–60 min) and when teams were losing. In such scenarios, teams regained possession higher up the pitch, employing more aggressive defensive strategies. Team quality emerged as the most decisive factor, with better-ranked teams displaying longer, more structured attacks and faster ball interventions to score. The findings suggest that success in elite soccer is driven not only by team quality, but also by adaptability to match-specific conditions. Integrating these situational factors into both training and match preparation is vital to developing a team’s adaptability to the ever-evolving contextual dynamics of elite soccer.

## Introduction

The global fascination with soccer (association football) is reflected in its widespread viewership and the marked expansion of performance analysis research over the past decade (Sarmento et al., 2022). Advanced automated and semi-automated video analysis and tracking systems have revolutionised the collection of vast, multidimensional information, making traditional notational methods less prominent but still crucial. While physical performance metrics continue to dominate research (Errekagorri et al., 2022; Otero-Saborido et al., 2024) providing valuable benchmarks for coaching and training (Bradley, 2024), there is an urgent need to focus on technical-tactical aspects that are pivotal in understanding match dynamics and outcomes (Barthelemy et al., 2024; Lago-Peñas et al., 2023; Konefał et al., 2019; Yan et al., 2024).

The use of match-aggregated statistics from data providers to examine emerging technical and tactical trends in soccer has gained prominence in sports science. Although whole- match data can grant a clear overview of team behaviour (Barthelemy et al., 2024; Lepschy et al., 2021), it may overlook critical, often invisible patterns, such as the mechanisms behind goal-scoring events (Almeida, 2019; Praça et al., 2024). Goals in soccer are rare and decisive episodes with a minimal probability of reoccurring in the same way (Kubayi and Larkin, 2022); yet, goal-scoring behaviours are not entirely random, suggesting the existence of patterns that drive goal creation (Anzer et al., 2021). Therefore, moving beyond the popular analysis of game-related statistics, notational analysis greatly contributes to a better understanding of the team patterns that lead to success in modern soccer (Almeida, 2019; Sarmento et al., 2022).

Some studies highlight that goal-scoring opportunities may better predict overall team performance than goals themselves (Aguado-Méndez et al., 2021; Schulze et al., 2022). Nevertheless, completed goal-scoring sequences offer essential insights into the technical-tactical and situational dynamics that culminate in goals and demonstrate the value of notational analysis for capturing these quality-driven actions. In this sense, recent literature underscores a shift towards attacking efficiency, where top teams prioritise quality over quantity in their offensive endeavours. These teams often leverage higher passing tempos and faster ball movements to effectively penetrate well-organised defences (Schulze et al., 2022; Taha and Ali, 2023; Wallace and Norton, 2014). This trend towards more dynamic and tactically astute playing styles favours effective possession over prolonged control, as groundbreakingly evidenced by Collet (2013). The emphasis on quality is further corroborated by studies linking competitive success (e.g., advancing to the FIFA World Cup knockout phase) to shooting accuracy, rather than shot volume (Degrenne and Carling, 2024; Errekagorri et al., 2022; Kubayi and Larkin, 2022).

Efficiency in the attacking phase extends beyond simply scoring goals, including how teams regain and maintain possession. Successful teams often disrupt opponents early by regaining possession higher up the pitch (Almeida et al., 2014; Santos et al., 2017), maintaining longer possessions near the opponent’s goal within constrained spaces (Iván-Baragaño et al., 2024). Evolving strategies also involve building up from the back, utilising goalkeepers and central defenders in playmaking roles (Pan et al., 2024). However, achieving positive outcomes in top-level tournaments requires not only efficiency and tight defensive coordination, but also the ability to adapt strategically and tactically to various match contexts (Aguado-Méndez et al., 2021; Yan et al., 2024). This insight underlines the imperative for soccer strategies to centre around improving the quality of team interactions in offensive play without being confined to a single approach (positional attack, fast attack or counterattack) and disregarding the competitive circumstances.

In this vein, a compelling body of evidence supports situational variables’ impact on attacking and defending performances in professional soccer (Almeida et al., 2014; Santos et al., 2017; Sarmento et al., 2018, 2022). Notwithstanding their relevance, much of the research conducted on FIFA Men’s World Cups has neglected critical variables such as *team/opponent quality, match period*, and *match status* (Kubayi and Larkin, 2022; Praça et al., 2024; Yan et al., 2024; Yi et al., 2019). This oversight discloses a notable gap in the literature, stressing the need for further studies that integrate these situational variables to better grasp their influence on elite-level soccer match performance.

Ongoing research has pointed out the central role of a team’s relative strength in differentiating among team performances at the highest levels of soccer. At the latest FIFA World Cup, better-ranked teams outperformed their lower-ranked counterparts in shooting and passing, displaying not only more attempts at the goal and shots on target, but also superior passing accuracy and possession control (Braquinho et al., 2024; de França et al., 2024; Pan et al., 2024). These disparities are often attributed to the stronger teams’ tendency to adopt a possession-based strategy, in contrast to the faster, counterattack-focused methods preferred by lower-ranked teams (Castellano et al., 2013; de França et al., 2024; Pan et al., 2024). Defensively, higher-ranked teams applied more aggressive and coordinated pressure in advanced field positions than lower-ranked ones (de França et al., 2024; Iván-Baragaño et al., 2024; Praça et al., 2024).

Despite the extensive data on the timing of goals, with a noteworthy increase during the second half and especially in the final period (76 min–full-time) (Degrenne and Carling, 2024; Kubayi and Toriola, 2019; Mićović et al., 2023), comprehensive perspectives into the mechanisms of goal production throughout the match remain limited (Sarmento et al., 2022). The existing research also indicates that different match statuses—reflected in the goal differences between teams—prompt distinct technical-tactical behaviours. However, the available data are still too sparse and heterogeneous to draw generalisable conclusions (Sarmento et al., 2022). Therefore, it is paramount to explore how various situational variables impact key performance indicators (KPIs) that characterise goal-scoring offensive sequences in elite soccer.

This study aimed to examine the impact of four situational variables, i.e., *competition stage, match period, match status*, and *team quality*, on offensive sequences leading to goals during the 2022 FIFA Men’s World Cup in Qatar. By integrating a comprehensive set of KPIs alongside these situational factors, this research sought to provide a finer-granular analysis of goal origins in this globally celebrated tournament (Branquinho et al., 2024; Iván-Baragaño et al., 2024; Yan et al., 2024). The findings were expected to enrich strategic and tactical preparations for the World Cup, offering valuable insights for refining team strategies and developing practical guidelines tailored specifically to the demands of this premier sporting event.

## Methods

### Sample

The sample consisted of all offensive sequences resulting in goals during the regular time (*n* = 168) in the 2022 FIFA World Cup held in Qatar. This prestigious tournament comprised two distinct stages: (1) the 32-team group stage, where national teams were divided into eight groups of four teams, each playing three matches against their pool opponents, in a total of 48 matches; and (2) the knockout phase that followed the group stage and consisted of single-elimination matches leading to the crowning of the world champion. The knockout phase included a round of 16, quarter-finals, semi-finals, play-off for the third place, and the final (totalling 16 matches).

The offensive sequences analysed in this study encompass goals scored during both the group stage and the knockout phase of the tournament. Due to the low number of instances, goals scored during extra-time—specifically, four goals from two matches (Croatia vs. Brazil in the quarter-final and Argentina vs. France in the final)—were excluded from the analysis. Match recordings were obtained from FIFA-authorised TV broadcasts and converted to MP4 format for further analysis. The study adhered to the Declaration of Helsinki’s recommendations and was approved by the Scientific Board of the Department of Psychology and Physical Education at the Manuel Teixeira Gomes Higher Education Institute (ISMAT), Portimão, Portugal (approval code: 004-23; approval date: 22 November 2023).

### Design

We employed a retrospective observational methodology to conduct a comparative analysis of goal-scoring offensive sequences. Following the observational methodology framework proposed by Portell et al. (2015), this study was classified as the sixth of eight possible types of observational study designs: multidimensional (considering multiple response levels, including quantitative and categorical variables), nomothetic (analysing multiple teams and matches), point/single (focusing on a single tournament edition), and extensive (using static performance indicators).

### Variables and Procedures

An offensive sequence, defined as a series of individual and/or collective actions leading to a goal, was delineated based on Almeida’s criteria (2019). To characterise the offensive sequences, we utilised the *Offensive Sequences Characterisation System* (OSCS), a notational analysis system encompassing both simple and composite performance indicators (Almeida, 2019). Composite indicators, combining two simple variables, offered broader insights into offensive play. The OSCS underwent validation by an external panel of experts, comprising two UEFA Pro coaches with top-tier European soccer experience and two independent sports science researchers with over a decade of expertise in performance analysis. For face and content validity, the experts reviewed the variables and operational definitions, provided feedback, and suggested refinements. After incorporating these changes, full consensus was reached and the system was validated. Detailed operational definitions for these quantitative dependent indicators can be found in Table 1.

**Table 1:** Performance-related dependent indicators and operational definitions in the *Offensive Sequences Characterisation System* (OSCS) (Almeida, 2019).

Performance Indicators	Operational definitions
Simple	
Duration of ball possession (s)	Total duration (in seconds) when the ball is in play in a given offensive sequence. Any interruption of the offensive sequence (e.g., foul or ball out) is not considered for the analysis.
Players involved (n)	Number of players that effectively played the ball (i.e., with on-the-ball actions) during the offensive sequence.
Ball touches (n)	Number of contacts with the ball, made with any (legal) part of the body, during the offensive sequence.
Passes (n)	Number of passes completed (i.e., ball intentionally played from one player to a teammate) during the offensive sequence.
Crosses (n)	Number of balls sent into the opposition team’s area from a wide position during the offensive sequence. Valid for actions performed from a lateral corridor, outside the penalty area and in the attacking third of the field.
Shots (n)	Number of attempts to score a goal, made with any (legal) part of the body, during the offensive sequence.
Set pieces (n)	Number of static situations deriving from opponents’ clearances, turnovers or fouls observed since the beginning until the end of the offensive sequence (goal kicks, throw-ins, corner kicks and free kicks). Note: if the offensive sequence starts with a set piece, the event is considered for the analysis.
Composite	
Players involved/Duration (n)	Tempo of collective involvement in the offensive sequence.
Ball touches/Duration (n)	Tempo of intervention on the ball in the offensive sequence.
Passes/Duration (n)	Tempo of ball transmission between teammates in the offensive sequence.
Ball touches/Players involved (n)	Measure of individual intervention on the ball in the offensive sequence.
Passes/Players involved (n)	Individual contribution to ball passing in the offensive sequence.
Passes/Ball touches (n)	Playing style adopted by teams in the offensive sequence (team-based vs. individual attacking strategies).
Goal/Shots (%)	Measure of shot effectiveness in the offensive sequence expressed as a percentage.

In addition to the performance indicators derived from the OSCS, three categorical variables were included as dependent variables: *ball recovery type, ball recovery location* (Almeida et al., 2014), and *team possession type* (Sarmento et al., 2018). The offensive sequences were further analysed based on four independent variables: *competition stage, match status, match period*, and *team quality*. Table 2 provides an overview of the type, categories, and operational definitions of each categorical variable included in the study.

**Table 2:** Categories, operational definitions, and collection procedures of categorical variables in the study.

Variable	Categories	Operational definition/collection procedures
Independent		
Competition stage	1) Group stage 2) Knockout phase	Indicates the stage of the competition during which the goal-scoring event occurred.
Match status	1) Losing 2) Tied 3) Winning	Represents the evolving score of a match immediately before the goal-scoring event. Categories were defined in relation to the number of goals scored and conceded by the scoring team at the time of data entry.
Match period	1) 1–30 min 2) 31–60 min 3) 61 min–FT	Recorded as 1, 2 or 3 depending on the period in which the goal was scored during the match (Almeida et al., 2014). Note: FT – full-time.
Team quality	1) Worse-ranked 2) Similarly-ranked 3) Better-ranked	Represents the quality difference between the scoring team and its opponent. Considering the FIFA men’s world ranking just before the competition (October 6, 2022), a k-means cluster analysis was conducted on total points for grouping teams into three quality categories. For example, if the scoring team was playing against an opponent from a lower quality group, it was categorised as “better-ranked”.
Dependent		
Ball recovery type (Almeida et al., 2014)	1) Interception	When the defender prevents a ball passed by an opponent from reaching its intended receiver by contacting the ball and keeping his own team in possession of the ball.
	2) Tackle	When the defender dispossesses the opponent of the ball through a physical challenge or defensive pressure.
	3) Goalkeeper save	When the goalkeeper prevents the opposing team from scoring a goal after any kind of shot, i.e., a kick, a header or any intended deflection of the ball toward a goal.
	4) Set piece	Static situations deriving from opponents’ misses or fouls (goal kicks, thrown-ins, off-sides, and free kicks), and opponents’ goals.
	5) Turnover won	When the defender collects, somewhere in the pitch, a ball lost (clearances or missed passes) by the opposing team.
Ball recovery location (Almeida et al., 2014)	1) Defensive 2) Defensive midfield 3) Offensive midfield 4) Offensive	Determined by dividing the pitch into 4 transverse zones with the same size.
Team possession type (Sarmento et al., 2018)	1) Counterattack	Rapid progression of the ball using a degree of imbalance from the ball recovery zone to the finishing zone. Ball circulation occurs more in depth than in width. Reduced number of passes (≤5). Reduced number of players intervening directly on the ball (≤4). Reduced time of the offensive sequence (<12s).
	2) Fast attack	Ball circulation is performed in width and depth with short and quick passes. Reduced number of passes (≤7). A maximum of 6 players with direct intervention on the ball. The sequence time has a maximum of 18 s.
	3) Positional attack	Opposing team displays a balanced defensive organisation. Ball circulation is performed more in width than in depth, predominantly with short passes. High number of passes (>7) and players involved (>6). Long offensive sequence duration (>18 s).

The notational analysis was conducted using VLC Media Player (VideoLAN^®^ Organisation, France) to review video footage of goal-scoring offensive sequences. Microsoft^®^ Excel 365 (Microsoft^®^ Corporation, USA) was used to manually code the quantitative and categorical performance variables selected to characterise each offensive sequence. Afterwards, the Excel database was exported to Statistical Package for the Social Sciences (SPSS^®^), version 28.0 (IBM^®^ Corporation, USA), for data analysis.

### Reliability

Reliability in using the OSCS and the categorical variables was assessed through intra- and inter-observer testing procedures. Three observers were involved in the reliability assessment: the author (Ob1) and two other previously trained observers (Ob2 and Ob3), also co-authors of the study. All observers had more than ten years of experience in performance analysis techniques. Prior to reliability assessments, all observers completed three pilot sessions, each coding ten randomly selected goal-scoring offensive sequences from the 2021/2022 UEFA Champions League. Discrepancies were reviewed after each session to harmonise interpretations and maintain uniform application of the OSCS criteria.

To assess intra-observer reliability, Ob1 completed a test-retest protocol with a six-week period separating both sessions to prevent potential learning effects. Twenty offensive sequences were randomly selected from the total sample (~12%) for notation in each session. For inter-observer reliability assessment, the three observers participated in a coding session where they independently notated the same set of offensive sequences. The intraclass correlation coefficient (ICC) and Weighted kappa (κ_w_) were calculated to evaluate intra- and inter-observer agreements. The ICC was primarily used for discrete performance variables, while κ_w_ was applied for categorical variables and discrete indicators with low counts. Table 3 displays the intra- and inter-observer reliability results.

**Table 3:** Intra- and inter-observer reliability for quantitative and categorical performance-related variables.

Reliability measure and performance-related variable		Intra-observer	Inter-observer		
		Ob1_test_–Ob1_retest_	Ob1–Ob2	Ob1–Ob3	Ob2–Ob3
Intraclass Correlation Coefficient (ICC)					
Duration		1.000	0.991	0.999	0.999
Players involved		0.997	0.982	0.995	0.997
Ball touches		1.000	0.990	0.999	1.000
Passes		0.999	0.993	0.998	0.995
Weighted Kappa (κ_w_)					
Ball recovery type		1.000	1.000	1.000	1.000
Ball recovery location		1.000	0.940	0.940	0.940
Crosses		1.000	0.808	1.000	0.808
Shots		1.000	0.875	0.875	0.875
Set pieces		1.000	0.851	1.000	0.851
Team possession type		0.917	0.767	0.841	0.923

The results for intra-observer reliability testing demonstrated an overall excellent level of agreement for both the ICC (>0.99) and κ_w_ (>0.91). For inter-observer reliability, ICC values were consistently high (>0.98), indicating excellent agreement among observers. The strength of agreement measured by κ_w_ ranged from “good” (*team possession type*, Ob1 and Ob2: κ_w_ = 0.77) to “very good” (κ_w_ > 0.8) (O’Donoghue, 2010).

### Statistical Analysis

The effects of *competition stage, match status, match period*, and *team quality* on offensive performance indicators were initially explored using descriptive statistics, including medians, interquartile ranges, and relative frequencies. Since the assumptions for applying parametric tests were violated, specifically the assumption of normality, non-parametric tests were implemented. Mann-Whitney U tests were conducted for the *competition stage* and Kruskal-Wallis tests for *match status, match period*, and *team quality*.

The effect sizes (ES) for Mann-Whitney tests and post-hoc pairwise comparisons of Kruskal-Wallis tests were calculated employing the following equation (1), as indicated by Field (2018):


$$r=\frac{Z}{\sqrt{N}}$$

where *r* was the ES estimate for the Mann-Whitney test or the pairwise comparison of Kruskal Wallis test, *Z* the z-score produced by SPSS and *N* the number of total observations on which *Z* was based.

The interpretation of ES followed the benchmarks proposed by Cohen (1992): small (*r* ≥ 0.1), medium (*r* ≥ 0.3), and large (*r* ≥ 0.5). For categorical variables, we conducted Chi-square tests of independence to evaluate their association with *ball recovery type, ball recovery location*, and *team possession type*. Adjusted standardised residuals (AR) were calculated to further investigate the relationship between independent and dependent categorical variables. Residuals were deemed significant if they fell beyond the ±2.0 range, indicating frequencies higher or lower than expected with 95% confidence. ES were calculated using Cramer’s *V* statistic and assessed based on Cohen’s benchmarks (1992) for different degrees of freedom. The degrees of freedom for Cramer’s *V* were determined as the smaller of (R−1) or (C−1), with R representing rows and C representing columns (Gravetter and Wallnau, 2013). Subsequently, multinomial logistic regression was employed to explore how factors influenced categorical performance-related variables (*ball recovery type, ball recovery location*, and *team possession type*), with “interception”, “defensive zone”, and “positional attack” serving as reference categories. A significance level (α) of 0.05 was established for all statistical analyses.

## Results

This section is divided into three subsections to enhance reader comprehension: simple performance indicators, composite performance indicators, and categorical performance variables.

### Simple Performance Indicators

Key metrics such as duration, players involved, touches, passes, crosses, shots, and set pieces were examined across *competition stage, match status, match period*, and *team quality*. Table 4 summarises the median (Md) and interquartile range (IQR) for these indicators, highlighting significant results.

**Table 4:** Descriptive statistics for simple performance indicators of goal-scoring offensive sequences in the 2022 FIFA World Cup, presented as Medians and Interquartile Ranges (Md (IQR)).

Independent Variable / Categories	Duration	Players	Touches	Passes	Crosses	Shots	Set Pieces
Competition Stage							
Group stage	24.5 (32)	6.0 (5)	19.5 (25)	6.0 (11)	0.0 (1)	1.0 (0)	1.0 (1)
Knockout phase	24.0 (38)	6.5 (4)	20.5 (28)	6.0 (9)	0.0 (1)	1.0 (0)	1.0 (2)
Match Status							
Losing	23.0 (31)	6.0 (5)	18.0 (25)	6.0 (8)	1.0 (1)	1.0 (0)	1.0 (2)
Tied	28.0 (35)	7.0 (4)	21.0 (29)	7.0 (12)	0.0 (1)	1.0 (0)	1.0 (1)
Winning	24.0 (40)	6.0 (5)	19.0 (29)	6.0 (10)	0.0 (1)	1.0 (0)	0.5 (1)
Match Period							
1–30 min	33.0 (48)	8.0 (4)	22.0 (39)	8.0 (16)	1.0 (1)	1.0 (0)	1.0 (2)
31–60 min	18.5 (34)	6.0 (6)	17.0 (29)	5.50 (11)	0.0 (1)	1.0 (0)	1.0 (1)
61 min–Full-time	24.0 (26)	6.0 (5)	19.0 (20)	6.0 (7)	0.0 (1)	1.0 (0)	1.0 (1)
Team Quality							
Worse-ranked	17.5* (31)	5.0* (4)	13.5* (19)	4.5* (7)	1.0 (1)	1.0 (0)	1.0 (2)
Similarly-ranked	22.0 (28)	5.0 (6)	14.0^ (22)	5.0 (8)	0.0 (1)	1.0 (0)	1.0 (1)
Better-ranked	29.0* (37)	8.0* (4)	24.0*^ (33)	8.0* (11)	0.0 (1)	1.0 (0)	1.0 (1)

Notes: * indicates a significant difference between worse- and better-ranked teams; ^ indicates a significant difference between similarly- and better-ranked teams

Mann-Whitney U tests showed no significant differences between the group stage and the knockout phase across performance indicators. ESs were generally trivial, except for set pieces (*r* = 0.14; small).

The Kruskal-Wallis tests did not reveal significant differences across different match statuses (losing, tied, and winning) and match periods (1–30 min, 31–60 min, 61 min–FT) for any performance indicators. Minimal ES further endorsed the lack of significant differences. However, there were significant differences between worse-, similarly, and better-ranked teams for several performance indicators. Worse-ranked teams had significantly shorter offensive sequence duration than better-ranked teams (*p* = 0.045; *r* = −0.22, small ES). Lower-ranked teams involved fewer players (*p* = 0.044; *r* = −0.22, small ES) and performed fewer ball touches (*p* = 0.002; *r* = −0.31, medium ES) and passes (*p* = 0.023; *r* = −0.23, small ES) in their successful offensive sequences compared with better-ranked teams. Also, when building up to score, similarly ranked teams performed fewer touches on the ball (*p* = 0.014; *r* = −0.25, small ES) than better-ranked teams.

### Composite Performance Indicators

Table 5 depicts composite performance indicators by *competition stage, match status, match period*, and *team quality*, outlining central tendencies, variability, and significance across different situational circumstances.

**Table 5:** Descriptive statistics for composite performance indicators of goal-scoring offensive sequences in the 2022 FIFA World Cup, presented as Medians and Interquartile Ranges (Md (IQR)).

Independent Variable / Categories	Players/ Duration	Touches/ Duration	Passes/ Duration	Touches/ Players	Passes/ Players	Passes/ Touches	Goal/ Shots
Competition Stage							
Group stage	0.25 (0.15)	0.81 (0.22)	0.27 (0.12)	3.23 (2.35)	1.00 (0.87)	0.33 (0.13)	100.0 (0.00)
Knockout phase	0.26 (0.16)	0.84 (0.20)	0.28 (0.12)	3.64 (2.71)	1.00 (0.69)	0.35 (0.17)	100.0 (0.00)
Match Status							
Losing	0.28 (0.18)	0.79 (0.31)	0.26 (0.08)	3.00 (2.40)	0.89 (0.65)	0.33 (0.13)	100.0 (0.00)
Tied	0.25 (0.16)	0.83 (0.18)	0.29 (0.11)	3.33 (2.35)	1.00 (0.80)	0.35 (0.14)	100.0 (0.00)
Winning	0.25 (0.13)	0.83 (0.26)	0.25 (0.13)	3.64 (2.55)	1.00 (0.84)	0.33 (0.17)	100.0 (0.00)
Match Period							
1–30 min	0.21 (0.21)	0.82 (0.15)	0.28 (0.14)	3.67 (3.15)	1.20 (1.08)	0.35 (0.15)	100.0 (0.00)
31–60 min	0.29 (0.21)	0.85 (0.28)	0.28 (0.11)	3.23 (2.50)	1.00 (0.68)	0.34 (0.17)	100.0 (0.00)
61 min–Full-time	0.25 (0.12)	0.79 (0.25)	0.25 (0.12)	3.25 (2.10)	1.00 (0.70)	0.32 (0.13)	100.0 (0.00)
Team Quality							
Worse-ranked	0.29 (0.15)	0.74* (0.27)	0.26 (0.11)	2.57* (1.71)	0.86* (0.65)	0.32 (0.17)	100.0 (0.00)
Similarly-ranked	0.25 (0.18)	0.80 (0.22)	0.28 (0.14)	3.00 (2.29)	1.00 (0.79)	0.33 (0.14)	100.0 (0.00)
Better-ranked	0.24 (0.14)	0.85* (0.25)	0.28 (0.10)	3.71* (2.61)	1.13* (0.94)	0.34 (0.12)	100.0 (0.00)

Notes: * indicates a significant difference between worse- and better-ranked teams; ^ indicates a significant difference between similarly- and better-ranked teams

Mann-Whitney U tests revealed no significant disparities between competition stages across composite performance indicators. No statistically significant differences were observed, though ES varied across composite performance indicators. Effect sizes ranged from trivial (Touches/Players, *r* = 0.002) to large (Touches/Duration, *r* = 0.079; Passes/Duration, *r* = 0.056; Passes/Players, *r* = 0.051), with medium-sized effects observed for Players/Duration (*r* = 0.033), Passes/Touches (*r* = 0.041), and Goals/Shots (*r* = 0.041). These variations may be attributed to random variability within groups.

*Match status* and *match period* did not produce differences across composite performance indicators. However, Kruskal-Wallis’ procedures uncovered important distinctions between worse- and better-ranked teams. As a means to achieve a goal, low-ranking teams employed offensive sequences with a slower tempo of intervention on the ball (Touches/Duration: *p* = 0.012; *r* = −0.26, small ES), and with fewer ball touches (Touches/Players: *p* = 0.006; *r* = −0.28, small ES) and passes per player involved (Passes/Players: *p* = 0.038; *r* = −0.23, small ES) compared with better-ranked teams.

### Categorical Performance Variables

Tables 6–8 provide the frequencies, Chi-square test details, and adjusted residuals for each dependent categorical variable.

**Table 6:** *Ball recovery type* distribution (absolute and relative frequencies) by *competition stage, match status, match period*, and *team quality*.

Independent Variable / Categories		Ball Recovery Type, n (%)								
		Interception		Tackle		Goalkeeper Save		Set Play		Turnover Won
Competition Stage										
Group stage		31 (25.8)		21 (17.5)		2 (1.7)		35 (29.2)		31 (25.8)
Knockout phase		11 (22.9)		8 (16.7)		2 (4.2)		20 (41.7)		7 (14.6)
Match Status										
Losing		11 (28.2)		7 (17.9)		1 (2.6)		11 (28.2)		9 (23.1)
Tied		16 (21.9)		13 (17.8)		1 (1.4)		28 (38.4)		15 (20.5)
Winning		15 (26.8)		9 (16.1)		2 (3.6)		16 (28.6)		14 (25.0)
Match Period										
1–30 min		6 (19.4)		5 (16.1)		0 (0.0)		10 (32.3)		10 (32.3)
31–60 min		12 (18.8)		17 (26.6) +		1 (1.6)		22 (34.4)		12 (18.8)
61 min–Full-time		24 (32.9) +		7 (9.6) −		3 (4.1)		23 (31.5)		16 (21.9)
Team Quality										
Worse-ranked		8 (22.2)		5 (13.9)		1 (2.8)		12 (33.3)		10 (27.8)
Similarly-ranked		9 (18.8)		7 (14.6)		2 (4.2)		19 (39.6)		11 (22.9)
Better-ranked		25 (29.8)		17 (20.2)		1 (1.2)		24 (28.6)		17 (20.2)

Note 1: no significant association between independent variables and the ball recovery type was found (p > 0.05); + higher observed frequency than expected; − lower observed frequency than expected Note 2: Cramer’s V values of 0.165 (competition stage), 0.091 (match status), 0.193 (match period), and 0.127 (team quality)

**Table 7:** Ball recovery location distribution (absolute and relative frequencies) by competition stage, match status, match period, and team quality.

Independent Variable / Categories		Ball Recovery Location, n (%)						
		Defensive		Defensive Midfield		Offensive Midfield		Offensive
Competition Stage								
Group stage		47 (39.2)		41 (34.2)		21 (17.5)		11 (9.2)
Knockout phase		22 (45.8)		13 (27.1)		7 (14.6)		6 (12.5)
Match Status								
Losing		10 (25.6) −		15 (38.5)		9 (23.1)		5 (12.8)
Tied		36 (49.3)		18 (24.7)		10 (13.7)		9 (12.3)
Winning		23 (41.1)		21 (37.5)		9 (16.1)		3 (5.4)
Match Period *								
1–30 min		11 (35.5)		14 (45.2)		5 (16.1)		1 (3.2)
31–60 min		23 (35.9)		15 (23.4)		15 (23.4)		11 (17.2) +
61 min–Full-time		35 (47.9)		25 (34.2)		8 (11.0)		5 (6.8)
Team Quality								
Worse-ranked		15 (41.7)		11 (30.6)		7 (19.4)		3 (8.3)
Similarly-ranked		18 (37.5)		13 (27.1)		10 (20.8)		7 (14.6)
Better-ranked		36 (42.9)		30 (35.7)		11 (13.1)		7 (8.3)

Note 1: * p < 0.05; + higher observed frequency than expected; − lower observed frequency than expected Note 2: Cramer’s V values of 0.093 (competition stage), 0.163 (match status), 0.199 (match period), and 0.103 (team quality)

**Table 8:** Team possession type distribution (absolute and relative frequencies) by competition stage, match status, match period, and team quality.

Independent Variable / Categories		Team Possession Type, n (%)				
Counterattack		Fast Attack		Positional Attack
Competition Stage						
Group stage		20 (16.7)		26 (21.7) −		74 (61.7)
Knockout phase		5 (10.4)		18 (37.5) +		25 (52.1)
Match Status						
Losing		7 (17.9)		10 (25.6)		22 (56.4)
Tied		10 (13.7)		19 (26.0)		44 (60.3)
Winning		8 (14.3)		15 (26.8)		33 (58.9)
Match Period						
1–30 min		4 (12.9)		6 (19.4)		21 (67.7)
31–60 min		12 (18.8)		21 (32.8)		31 (48.4) −
61 min–Full-time		9 (12.3)		17 (23.3)		47 (64.4)
Team Quality						
Worse-ranked		7 (19.4)		11 (30.6)		18 (50.0)
Similarly-ranked		9 (18.8)		16 (33.3)		23 (47.9)
Better-ranked		9 (10.7)		17 (20.2)		58 (69.0) +

Note 1: no significant association between independent variables and the team possession type was found (p > 0.05); + higher observed frequency than expected; − lower observed frequency than expected Note 2: Cramer’s V values of 0.168 (competition stage), 0.035 (match status), 0.120 (match period), and 0.147 (team quality)

The Chi-square analysis revealed no significant associations between the independent variables and *ball recovery type* (*p* > 0.05). Despite the lack of statistical significance, small associations were generally observed. Set plays were the most common recovery type during the group stage (29.2%) and the knockout phase (41.7%). Teams in a losing position recovered the ball most frequently through interceptions (28.2%), while set plays were dominant when the match was tied (38.4%) or the team was winning (28.6%). In the first 30 min, set plays (32.3%) and turnovers won (32.3%) were prevalent. Between 31 and 60 min, tackles (26.6%) were higher than expected (AR = 2.5), whereas in the last third of the match interceptions (32.9%) were remarkably higher (AR = 2.1), and tackles (9.6%) were lower than expected (AR = −2.3). Better-ranked teams primarily recovered the ball through interceptions (29.8%) and set plays (28.6%). Worse-ranked teams had more recoveries through set plays (33.3%).

Table 9 presents the variable estimates for *ball recovery type*. The logistic regression model confirmed the influence of *match period* on the dependent variable. In short, the odds of regaining possession through a tackle instead of an interception significantly increased by 398.4% (*p* = 0.006) in the middle of matches relative to the last 30 min (plus additional time). The situational factors of *competition stage, match status*, and *team quality* did not have significant effects (*p* > 0.05).

**Table 9:** Variable estimates for the multinomial logistic regression of *ball recovery type* as a function of situational factors in FIFA World Cup 2022.

Variables / Categories			*B* (SE)	95% CI for Odds Ratio		
Lower	OR	Upper
Tackle (reference: Interception)						
Intercept*			−1.397 (0.697)			
Competition Stage: Group stage vs. Knockout phase			0.093 (0.568)	0.361	0.870	3.341
Match Status:		Losing vs. Winning	0.037 (0.774)	0.228	1.038	4.727
		Tied vs. Winning	0.002 (0.622)	0.296	1.002	3.391
Match Period:		1–30’ vs. 61’–FT	1.085 (0.793)	0.625	2.961	14.018
		31–60’ vs. 61’–FT**	1.606 (0.588)	1.576	4.984	15.763
Team Quality:		Worse vs. Better-ranked	0.104 (0.784)	0.238	1.109	5.161
		Similarly vs. Better-ranked	0.221 (0.638)	0.358	1.248	4.354
Goalkeeper Save (reference: Interception)						
Intercept			5.862 (0.785)			
Competition Stage: Group stage vs. Knockout phase			−1.952 (1.289)	0.033	0.323	3.189
Match Status:		Losing vs. Winning	−1.429 (1.568)	0.011	0.240	5.183
		Tied vs. Winning	−0.315 (1.473)	0.041	0.730	13.087
Match Period:		1–30’ vs. 61’–FT	−18.879 (0.001)	6.325E-9	6.325E-9	6.325E-9
		31–60’ vs. 61’–FT	−0.606 (1.279)	0.044	0.546	6.695
Team Quality:		Worse vs. Better-ranked	1.840 (1.746)	0.206	6.295	192.701
		Similarly vs. Better-ranked	1.962 (1.338)	0.516	7.111	97.927
Set Play (reference: Interception)						
Intercept			−0.116 (0.543)			
Competition Stage: Group stage vs. Knockout phase			−0.504 (0.472)	0.240	0.604	1.523
Match Status:	Losing vs. Winning		−0.534 (0.674)	0.156	0.587	2.198
	Tied vs. Winning		0.393 (0.532)	0.522	1.481	4.203
Match Period:	1–30’ vs. 61’–FT		0.298 (0.653)	0.375	1.347	4.846
	31–60’ vs. 61’–FT		0.523 (0.487)	0.650	1.687	4.379
Team Quality:	Worse vs. Better-ranked		0.868 (0.656)	0.658	2.382	8.619
	Similarly vs. Better-ranked		1.003 (0.532)	0.961	2.727	7.736
Turnover Won (reference: Interception)						
Intercept			−0.992 (0.636)			
Competition Stage: Group stage vs. Knockout phase			0.603 (0.566)	0.602	1.827	5.542
Match Status:	Losing vs. Winning		−0.850 (0.727)	0.103	0.427	1.777
	Tied vs. Winning		−0.592 (0.587)	0.175	0.553	1.747
Match Period:	1–30’ vs. 61’–FT		1.215 (0.674)	0.899	3.369	12.626
	31–60’ vs. 61’–FT		0.542 (0.541)	0.595	1.719	4.968
Team Quality:	Worse vs. Better-ranked		1.145 (0.710)	0.782	3.142	12.625
	Similarly vs. Better-ranked		0.802 (0.586)	0.707	2.229	7.027

Model χ^2^(28) = 29.440, p = 0.391. Pseudo R^2^ = 0.161 (Cox & Snell), 0.170 (Nagelkerke), 0.061 (McFadden) Note: * p ≤ 0.05; ** p ≤ 0.01; *** p ≤ 0.001

Furthermore, a significant association was found between *match period* and *ball recovery location* (*p* = 0.038). During the first 30 min, the defensive midfield (45.2%) was the most common recovery zone. Between 31 and 60 min, offensive recoveries (17.2%) were higher than expected (AR = 2.4). In the last 30 min, the defensive zone (47.9%) recoveries were prominent. The Chi-square analysis indicated no significant associations between the other independent variables and *ball recovery location* (*p* > 0.05); nonetheless, some patterns emerged, suggesting trivial (*competition stage*) to small associations (*match status* and *team quality*). The defensive zone was the most common recovery area in both the group stage (39.2%) and the knockout phase (45.8%), when matches were tied (49.3%) or the scoring team was winning (41.1%). When the score was unfavourable (losing), the ball recovery frequency in the defensive zone (25.6%) was lower than expected (AR = −2.2), occurring most regularly in the defensive midfield (38.5%).

The multinomial logistic regression model (Table 10) also indicated that *match status* and *match period* significantly affected the *ball recovery location*. The probability of recovering the ball preceding a goal scored in the defensive midfield, rather than the defensive zone, was 63.1% (*p* = 0.04) lower with an equalising score, compared to when ahead. On the contrary, when losing, teams increased by 530.6% (*p* = 0.045) the likelihood of regaining the ball in the offensive zone (vs. defensive one) compared to when winning. Moreover, the chances of regaining a goal-scoring possession in the defensive midfield (vs. defensive) significantly increased by 252.9% (*p* = 0.025) in the first third of the match compared to the final third. Similarly, there were increases of 321.3% (*p* = 0.009) and 301.2% (*p* = 0.033) in recovering the ball in the offensive midfield and offensive zones, respectively, rather than in the defensive one, between 31 and 60 min in relation to the last match period.

**Table 10:** Variable estimates for the multinomial logistic regression of *ball recovery location* as a function of situational factors in FIFA World Cup 2022.

Variables / Categories			*B* (SE)	95% CI for Odds Ratio		
				Lower	OR	Upper
Defensive Midfield (reference: Defensive)						
Intercept			−0.594 (0.490)			
Competition Stage: Group stage vs. Knockout phase			0.661 (0.435)	0.826	1.936	4.539
Match Status:		Losing vs. Winning	0.818 (0.630)	0.659	2.265	7.790
		Tied vs. Winning*	−0.998 (0.485)	0.142	0.369	0.954
Match Period:		1–30’ vs. 61’–FT*	1.261 (0.564)	1.168	3.529	10.661
		31–60’ vs. 61’–FT	0.238 (0.454)	0.521	1.269	3.088
Team Quality:		Worse vs. Better-ranked	−0.571 (0.605)	0.173	0.565	1.850
		Similarly vs. Better-ranked	−0.347 (0.470)	0.281	0.707	1.777
Offensive Midfield (reference: Defensive)						
Intercept***			−2.273 (0.687)			
Competition Stage: Group stage vs. Knockout phase			0.654 (0.536)	0.672	1.924	5.505
Match Status:		Losing vs. Winning	0.978 (0.736)	0.629	2.660	11.246
		Tied vs. Winning	−0.722 (0.604)	0.149	0.486	1.586
Match Period:		1–30’ vs. 61’–FT	1.345 (0.745)	0.892	3.839	16.519
		31–60’ vs. 61’–FT**	1.438 (0.551)	1.430	4.213	12.407
Team Quality:		Worse vs. Better-ranked	0.060 (0.699)	0.270	1.062	4.182
		Similarly vs. Better-ranked	0.445 (0.555)	0.526	1.561	4.631
Offensive (reference: Defensive)						
Intercept***			−2.956 (0.904)			
Competition Stage: Group stage vs. Knockout phase			−0.117 (0.614)	0.267	0.890	2.965
Match Status:	Losing vs. Winning*		1.842 (0.924)	1.031	6.306	38.590
	Tied vs. Winning		0.889 (0.786)	0.521	2.432	11.359
Match Period:	1–30’ vs. 61’–FT		−0.498 (1.217)	0.056	0.608	6.604
	31–60’ vs. 61’–FT*		1.389 (0.652)	1.118	4.012	14.403
Team Quality:	Worse vs. Better-ranked		−0.504 (0.847)	0.115	0.604	3.178
	Similarly vs. Better-ranked		0.690 (0.668)	0.538	1.993	7.383

Model χ^2^(21) = 35.894, p = 0.022. Pseudo R^2^ = 0.192 (Cox & Snell), 0.209 (Nagelkerke), 0.085 (McFadden) Note: * p ≤ 0.05; ** p ≤ 0.01; *** p ≤ 0.001

Although no significant associations were unveiled between the independent variables and *team possession type* (*p* > 0.05), there were observable trends with practical significance. The associations were classified as trivial for *match status* and small for *competition stage, match period*, and *team quality*. Positional attacks were the dominant possession type across all categories, particularly for better-ranked teams, where the observed frequency exceeded the expected value (AR = 2.7). In the 31–60-min period, there was a lower frequency of positional attacks than expected (AR = −2.2). Fast attacks became more prominent during the knockout phase (AR = 2.1) compared to the group stage, where the observed frequency was lower than anticipated (AR = −2.1). Counterattacks were the least common possession type overall, but were slightly more frequent for worse-ranked teams (19.4%).

The multinomial logistic regression analysis further confirmed the absence of significant effects of situational factors on *team possession type* (Table 11). Notably, the models explained a small portion of the variance in *team possession type* and a moderate portion in *ball recovery type* and *location*.

**Table 11:** Variable estimates for the multinomial logistic regression of *team possession type* as a function of situational factors in FIFA World Cup 2022.

Variables / Categories		*B* (SE)	95% CI for Odds Ratio		
			Lower	OR	Upper
Fast Attack (reference: Counterattack)					
Intercept		1.460 (0.748)			
Competition Stage: Group stage vs. Knockout phase		−1.101 (0.600)	0.102	0.332	1.078
Match Status:	Losing vs. Winning	−0.184 (0.775)	0.182	0.832	3.798
	Tied vs. Winning	0.322 (0.651)	0.385	1.380	4.938
Match Period:	1–30’ vs. 61’–FT	−0.511 (0.823)	0.120	0.600	3.009
	31–60’ vs. 61’–FT	−0.250 (0.571)	0.255	0.779	2.383
Team Quality:	Worse vs. Better-ranked	−0.049 (0.744)	0.221	0.952	4.093
	Similarly vs. Better-ranked	−0.006 (0.607)	0.302	0.994	3.271
Positional attack (reference: Counterattack)					
Intercept***		2.393 (0.692)			
Competition Stage: Group stage vs. Knockout phase		−0.375 (0.570)	0.225	0.688	2.101
Match Status:	Losing vs. Winning	0.298 (0.697)	0.343	1.347	5.285
	Tied vs. Winning	0.335 (0.588)	0.442	1.398	4.425
Match Period:	1–30’ vs. 61’–FT	−0.208 (0.707)	0.203	0.812	3.244
	31–60’ vs. 61’–FT	−0.871 (0.519)	0.151	0.418	1.158
Team Quality:	Worse vs. Better-ranked	−1.129 (0.675)	0.086	0.323	1.215
	Similarly vs. Better-ranked	−1.015 (0.554)	0.122	0.362	1.073


Model χ^2^(14) = 18.515, p = 0.184. Pseudo R^2^ = 0.104 (Cox & Snell), 0.123 (Nagelkerke), 0.058 (McFadden) Note: * p ≤ 0.05; ** p ≤ 0.01; *** p ≤ 0.001

## Discussion

Research on performance analysis, especially match analysis, has significantly advanced over the past decade, driven by emerging technologies and the proliferation of data providers (Sarmento et al., 2022). However, numerous scholars have emphasised the need for more focused studies on technical-tactical performance indicators while accounting for regularly overlooked situational variables (Barthelemy et al., 2024; Lago-Peñas et al., 2023; Praça et al., 2024; Yan et al., 2024). Addressing this gap, this study examined the impact of *competition stage, match period, match status*, and *team quality* on KPIs characterising goal-scoring offensive sequences during the 2022 FIFA World Cup.

The main findings illuminate the influence of situational variables on successful offensive sequences in elite soccer. *Team quality* proved pivotal, as better-ranked teams exhibited more complex and prolonged offensive actions than their lower-ranked counterparts. Surprisingly, variables such as *competition stage, match period*, and *match status* did not significantly alter simple and composite performance indicators. This suggests that teams maintained a consistent strategic-tactical approach during the build-up and progression towards scoring. Nonetheless, the analysis revealed that *match period* and *match status* affected both the manner and the location of possession regains critical to setting up goals. Though *team quality* was the dominant factor in determining the effectiveness of offensive strategies, there was a subtle interaction between the match context and team performance that predominantly influenced defence-to-offence transition behaviours as matches unfold.

Since the 2018 World Cup, distinguishing between teams eliminated at the group stage and those progressing to the knockout phase has become a common method for analysing team performance (Iván-Baragaño et al., 2024; Kubayi and Larkin, 2022; Yi et al., 2019). Although this approach represents a useful proxy for team strength, it neglects the varied competitive demands characteristic of each tournament stage. Our findings revealed no significant performance differences between the group and knockout stages, suggesting that elite national teams were well-prepared to manage both the pressures and elevated stakes of knockout rounds (Fernandes et al., 2020). However, the observed variation in effect sizes across different KPIs signalled complex performance dynamics not fully captured by median comparisons alone. In the knockout phase, there was a meaningful increase in fast attacks, accompanied by an increment in the tempo of ball interventions and collective involvement in goal-scoring events. The trend towards quicker, more aggressive possession styles likely represents a tactical adaptation to better exploit unusual imbalances in opponents’ defensive organisation (Almeida, 2019; Sarmento et al., 2018).

At the match level, segmenting full-time matches into smaller intervals has been widely recognised as a crucial situational factor in soccer performance analysis, with numerous studies stressing its significant impact on various performance metrics (Sarmento et al., 2022). A higher incidence of goals towards the end of matches (76 min–FT) has been frequently noted, likely due to fatigue accumulation (Aguado-Méndez et al., 2020; Degrenne and Carling, 2024; Kubayi and Toriola, 2019; Mićović et al., 2023). This observation suggests that teams might shift their strategic-tactical approaches as matches draw to the final whistle.

Despite the absence of significant changes in simple and composite performance indicators with match progression, categorical variables remained sensitive to timing, pointing to intricate dynamics between *match period* and performance. During the mid-game (31–60 min), teams markedly adjusted their defensive tactics to initiate successful offensive sequences. The increased defensive aggressiveness, demonstrated by an increase in ball recoveries through tackles, coincided with shifts in *ball recovery location*, moving from defensive midfield (early game) to more offensive zones (mid-game). Aguado-Méndez et al. (2021) similarly reported a threefold increase in the likelihood of conceding a goal-scoring opportunity due to an opponent’s steal, rather than a turnover, during the second half, which underscores the importance of defensive pressure as play evolves. Furthermore, this aggressive defence facilitated the employment of quicker attacking styles, such as fast attacks instead of positional play, seemingly exploiting opponents’ disarray during transitions to breach less organised defensive lines (Aguado-Méndez et al., 2020, 2021; Lago-Ballesteros et al., 2012; Sarmento et al., 2018). As matches approached the end, there was a noticeable increase in goal-scoring sequences originating from the defensive zone. This may reflect teams’ ability to maintain tight formations and exploit opportunities while arguably defending a favourable scoreline.

Transitioning from the influence of match progression, it is paramount to explore how *match status* (winning, tied or losing) affects the successful technical-tactical behaviours of high-level teams. Notwithstanding the multiple existing research insights, the data on this relationship remain mixed and inconclusive (Sarmento et al., 2022). Our analysis uncovered no significant differences in simple and composite performance indicators based on the scoreline, contrasting with prior studies positing that teams behind in a match tend to increase possession and passing frequency to control the game (Konefał et al., 2018, 2019; Lago, 2009; Lago-Ballesteros et al., 2012). Instead, teams in the 2022 FIFA World Cup maintained a consistent attacking approach aimed at scoring, regardless of the evolving *match status*. Perhaps the exclusive focus on goal-scoring offensive sequences reduces the sensitivity needed to detect fine-grained changes occurring during match-status maintenance or transition phases (Aguado-Méndez et al., 2021; Konefał et al., 2019; Schulze et al., 2022). A broader analytical approach may better capture the tactical and behavioural adjustments teams make in response to the current score. Nonetheless, we detected a significant effect of *match status* on *ball recovery location*: teams were more likely to recover possession in offensive zones when losing, a trend also corroborated in previous research (Almeida et al., 2014; Santos et al., 2017). This shift conceivably reflects tactical adjustments, such as pushing defensive lines higher and applying aggressive pressing to regain possession and exploit vulnerabilities. Heightened urgency after conceding may also lead players to take greater risks in creating scoring opportunities from these offensive recoveries (Santos et al., 2017).

Unlike Lago (2009), who emphasised the evolving scoreline as the critical variable influencing possession and play zones, our findings corroborate those of Castellano et al. (2013), which pinpoint *team quality* as the most influential factor in successful offensive sequences. Both quantitative performance indicators and categorical variables demonstrated a clear tendency for better-ranked teams to adopt a more controlled and structured approach when building from the back and progressing into the final third to score. Such structured play is often linked to team spatial distribution, with higher offensive width, length, and surface area observed in the offensive phase against weaker opponents (Castellano et al., 2013). In practice, this resulted in longer sequences, greater player involvement, and more touches and passes compared to lower-ranked teams (Zhou et al., 2019). These results uphold prior findings from the same tournament edition (Branquinho et al., 2024; de França et al., 2024; Pan et al., 2024), where stronger teams favoured positional attacks to score. Of note, these teams improved their efficiency by increasing the tempo of ball intervention and involving more players in passing connections (Pan et al., 2024; Taha and Ali, 2023; Yi et al., 2019). Interestingly, during defence-to-attack transitions, and contrasting previous studies (Iván-Baragaño et al., 2024; Praça et al., 2024; Santos et al., 2017), better-ranked teams did not recover possession more often in advanced pitch zones to create goal-scoring events. Rather, their attacking success relied less on where possession was regained and more on how they moved the ball once in control.

## Limitations and Future Research

Some limitations must be considered when interpreting the findings. First, the analysis focused solely on goal-scoring sequences, which represent the least frequent type of attacking plays in a match. Consequently, the data may not fully capture the broader spectrum of offensive dynamics (Aguado-Méndez et al., 2021; Schulze et al., 2022). Future research should expand the sample to include non-successful offensive sequences thereby providing a more holistic understanding of team performance. Additionally, due to the relatively small sample size, the *match period* and *match status* variables were consolidated into fewer categories, theoretically limiting the depth of the analysis. More extensive studies could mitigate this limitation by using a more granular segmentation of both variables. Second, whereas *team quality* was assessed using FIFA’s rankings before the tournament via K-means clustering, this may not entirely reflect a team’s strength or consistency throughout the World Cup. Future research could explore models that account for fluctuations in team performance over the competition. Third, the tool used in this study relied on static variables rather than sequential ones, restricting the ability to examine the complex and dynamic nature of soccer. Upcoming studies should incorporate sequential analyses, such as time-series, lag sequential, or t-pattern approaches, to more accurately depict behavioural changes throughout matches. Lastly, improving the multinomial logistic regression models with larger datasets from multiple World Cup editions would enhance the robustness and generalisability of the findings, yielding deeper insights into the evolving trends of elite soccer.

## Practical Implications

From the study’s main findings, we propose four practical applications to help coaching staff enhance performance in high-level tournaments:

1. Teams can analyse performance metrics in finer detail to identify strategic shifts, such as increasing dynamic ball movement and player rotations, to improve outcomes against stronger defences in later stages of the match.
2. Coaches may adopt an ecological approach to foster adaptive defensive strategies, encouraging increased assertiveness as the match progresses. Training should focus on recovering the ball in advanced areas to facilitate faster attacks by exploiting weaknesses in the opponent’s defensive transitions.
3. Manipulating *match status* during training, along with other task constraints (e.g., numerical relation), can simulate real-game scenarios, promoting higher defensive lines and aggressive pressing to regain possession near the opponent’s goal.
4. Adaptive attacking strategies based on relative team strength are paramount. Lower-ranked teams might focus on increasing the speed and efficiency of ball movement. At the same time, higher-ranked sides can optimise controlled, structured play, balancing positional and faster styles to suit different match phases.

## Conclusions

This study stresses the crucial role situational variables play in shaping goal-scoring sequences in elite soccer, with *team quality* emerging as the most decisive factor. Better-ranked teams demonstrated longer, more structured attacks with a greater tempo of ball intervention. Although *competition stage*, *match period*, and *match status* did not substantially impact KPIs related to build-up and progression, they influenced defensive-to-offensive transitions, especially in mid-game and when teams were behind. Ultimately, elite soccer’s success is driven by both *team quality* and situational adaptability. To optimise performance under varying match circumstances, coaches must account for these variables in training and match preparations, as fostering a team’s adaptability to the ever-changing contextual dynamics is fundamental to succeed in modern soccer.
